# Palliative Liver Radiotherapy (RT) for Symptomatic Hepatocellular Carcinoma (HCC)

**DOI:** 10.1038/s41598-020-58108-1

**Published:** 2020-01-27

**Authors:** Cynthia S. Y. Yeung, C. L. Chiang, Natalie S. M. Wong, S. K. Ha, K. S. Tsang, Connie H. M. Ho, B. Wang, Venus W. Y. Lee, Mark K. H. Chan, Francis A. S. Lee

**Affiliations:** 1 Department of Clinical Oncology, Tuen Mun Hospital, Tuen Mun, Hong Kong (SAR) China; 2 Department of Clinical Oncology, University of Hong Kong, Pok Fu Lam, Hong Kong (SAR) China; 3 Department of Clinical Oncology, HKU-Shenzhen Hospital, ShenZhen, China; 4 Department of Radiation Physics, Imperial College London NHS Healthcare Trust, Charing Cross Hospital, London, UK

**Keywords:** Outcomes research, Radiotherapy

## Abstract

This study aims at evaluating the symptom response, response duration, and toxicity of single dose palliative liver radiotherapy (RT) for symptomatic HCC patients. We reviewed unresectable HCC patients treated with palliative RT in our institution. Eligible patients were unsuitable or refractory to trans-arterial chemoembolization (TACE) and stereotactic body radiotherapy (SBRT), with an index symptom of pain or abdominal discomfort. The primary outcome was the percentage of patients with clinical improvement of index symptom at 1 month. Secondary outcomes were response duration, toxicities, alpha-feto protein (AFP) response, and radiological response. Fifty-two patients were included in the study. The index symptom was pain in 34 patients (65.4%), and abdominal discomfort (34.6%) in 18 patients. At 1 month, 51.9% of patients had improvement of symptoms. Median time to symptom progression was 89 days (range: 12–392 days). Treatment was well tolerated with only 2 patients (3.8%) developing grade 3 GI toxicities. AFP response, radiological response rate, and disease control rate at 3 months were 48.6%, 15.1%, and 54.5% respectively. Half of the patients had improvement of index symptoms after receiving palliative liver RT with median response duration of 3 months. The treatment was well tolerated with minimal toxicities.

## Introduction

Hepatocellular carcinoma (HCC) is the leading cause of global cancer death^1^. Surgical interventions provide the only chance of cure, yet majority of patients present with advanced disease and are treated with trans-arterial chemoembolization (TACE), targeted therapy, or best supportive care (BSC).

Conformal high dose radiotherapy (RT) or stereotactic body radiotherapy (SBRT) has emerged as a promising local therapy in treating unresectable HCC. Multiple studies demonstrated the tumor control at 1 year and 2 year in majority of patients^2–4^. Yet, because of the extensive liver involvement with cancer, borderline liver function, and/or presence of extra-hepatic metastases, many patients are not suitable for high dose radiation. The tolerance of whole liver to radiation is low^5^.

Patients who present in advanced stage often experience symptoms of pain or abdominal discomfort at some point of their illnesses^6^. Effective palliation of hepatic pain is challenging. To date, there is limited research regarding palliative radiotherapy (RT) specifically in the HCC population. In the early studies of whole-liver RT in patients mainly with liver metastases at doses ranging from 20–30 Gy, benefit of symptom palliation was observed in 49–95% of patients with limited toxicities^7–14^. Until recently, in a prospective phase II trial of 41 patients with HCC and liver metastases, the use of 8 Gy in single fraction to the whole liver resulted in a clinically meaningful improvement in average index symptom intensity in 48% of patients one month following RT. Benefits were only observed in patients with pain and abdominal discomfort. Treatment was well tolerated with only one patient developed grade 3 toxicity^15^. The randomized controlled trial to compare palliative RT against BSC in terminal HCC patients is underway.

The use of 8 Gy single fraction RT is attractive for HCC patients with short life expectancy. Yet, there has been limited published experience on the efficacy and safety of such treatment. In our institution, palliative liver RT is indicated in Barcelona Clinic Liver Cancer (BCLC) advanced or terminal stage (BCLC stage C and D) patients with tumor causing hepatic pain or abdominal discomfort. This study aims at evaluating the symptom response, response duration, and toxicity of palliative liver RT in symptomatic HCC patients.

## Materials and Methods

### Eligibility

In this study, which was approved by the Institutional Review Board of the University of Hong Kong/Hospital Authority Hong Kong West Cluster (HKU/HA HKW IRB) (IRB number: NTWC/CREC/18044), were performed in accordance with the relevant guidelines and regulations. We reviewed outcomes of 52 patients who received palliative RT (8 Gy single fraction) for HCC between July 2012 and December 2017 in our hospital. According to our institutional protocol, eligible patients of treatment had HCC diagnosed pathologically or by American Association for the Study of Liver Disease (AASLD) criteria who were unsuitable or refractory to TACE, resection, transplantation, radiofrequency ablation (RFA), or ablative dose of SBRT. All of them had index symptoms of either pain or abdominal discomfort. Other eligibility criteria includes, Eastern Cooperative Oncology Group (ECOG) performance status of 0 to 3, expected survival ≥ 1 month, platelet count > 25 × 10^9^/L, hemoglobin > 70 g/L, international normalized ratio (INR) < 3, bilirubin < 100 umol/L, and AST and ALT < 10 x upper limit of normal (ULN). Patients with child-pugh (CP) score ≥ C10, prior RT or selective internal radiation therapy (SIRT) to abdomen, targeted therapy or systemic therapy received within 2 weeks prior radiotherapy, or TACE received within 4 weeks prior RT would be considered to be ineligible.

### Treatment

All patients were pre-medicated with 5HT3 antagonist and oral dexamethasone 4 mg daily on the date of RT. Patients who were hepatitis B carriers were pre-emptively started on entecavir 0.5 mg daily at least one week before RT.

At RT planning, patients were simulated in a supine position using VacLok (MEDTEC, Iowa, USA) with a non-contrast computed tomography (CT) scan. Free-breathing during simulation CT was allowed. The gross tumor volume (GTV) includes all HCC foci causing pain or abdominal discomfort; delineation of GTV was aided by co-registration with diagnostic tri-phasic contrast CT. The minimum margin of 0 mm was added around GTV to form the clinical target volume (CTV). The CTV consisted of (almost) whole liver in patients with diffuse tumor involvement. The planning target volume (PTV) was generated from the CTV with a 5 mm axial margin and 10 mm cranial caudal margin to account for setup error and organ motion.

The prescription dose of 8 Gy in a single fraction was chosen based on the prospective phase II study from the Princess Margaret Cancer Center Group^14^. Treatment was planned with either a 6MV or 10MV photons using the conformal technique. PTV was aimed to receive 95% to 107% of the prescribed dose with adherence to the International Commission on Radiation Units & Measurements (ICRU) 62 report. Maximum dose to the stomach, small bowel, and spinal cord had to be less than 10 Gy.

### Patient assessment

Clinicians prospectively followed patients according to the institutional protocol. Symptoms, toxicities, liver function and alpha-feto protein (AFP) were monitored at baseline, week 2, week 4, monthly in the first 6 months after RT, and every 3 months thereafter till death. The use of analgesic and other medications were also recorded at each assessment. Physician reported symptoms were classified into 4 categories: complete response (CR), partial response (PR), stable disease (SD), and progressive disease (PD) which were defined as follows (13–14): CR: complete disappearance of index symptom and not requiring any regular analgesics; PR: partial improvement of index symptom without an increase in consumption of analgesics; SD: stable index symptom without increase in consumption of analgesics; PD: either worsening of index symptom or an increase in consumption of regular analgesics. Potential toxicities were graded using the National Cancer Institute Common Terminology Criteria for Adverse Events (CTCAE) version 4.0. Liver function deterioration was defined by worsening of CP score ≥ 2 at three months of RT completion. Liver toxicity was censored at the time of intrahepatic progression. Radiological assessment using computed tomography (CT) was not mandatory and at the discretion of physicians. The tumor response was measured using Response Evaluation Criteria In Solid Tumors (RECIST) criteria version 1.1.

### Statistics

The primary endpoint was the percentage of patients with clinical improvement of index symptom (CR + PR) at 1 month. Secondary endpoints were symptom response duration, toxicities, overall survival (OS), AFP response, and radiological response. Symptom response duration was defined as the period from date of RT to the date of index symptom deterioration. OS was calculated from the date of RT to the date of death due to whatever causes. AFP response was defined as > 20% drop from baseline^16^.

R version 3.25 (Vienna, Austria) was used for statistical analysis. Symptom response, response duration, radiological and AFP response, and toxicities were summarized with descriptive statistics. Comparison of categorical data between groups was performed by Chi-Square test or fisher exact test if appropriate. A Kaplan-Meier curve was used to calculate the OS. The log-rank test was used to compare outcomes among survival curves for potential prognostic factors. Any factor that was significant in univariate analyses was subjected to multivariate analyses using the Cox proportional hazards regression model. A statistical level of p = 0.05 was considered significant.

## Results

### Patients and treatment

From July 2012 to December 2017, a total of 52 patients fell under the aforementioned eligible criteria were treated and prospectively followed in accordance to our institutional protocol. Baseline patient and treatment characteristics are presented in Table 1. 38 patients (73%) were hepatitis B carriers. Median size of the largest tumor was 13 cm (range, 3–24 cm); 33 patients (63.5%) had tumor involvement > 50% of total liver volume; majority (98.1%) had BCLC stage C disease. 34 patients (65.4%) received no prior treatment. 23 patients (45.1%) were treated with Sorafenib after RT, while half of them received no further treatment.

**Table 1:** Patient Demographics and Clinical Characteristics (N = 52).

Characteristics	No of patients (%)
Age at diagnosis, years	
Median	60
Range	37–87
Sex	
Male	44 (84.6%)
Female	8 (15.4%)
ECOG performance status	
0	5 (9.6%)
1	32 (61.5%)
2	15 (28.9%)
3	0 (0.0%)
Time from diagnosis to RT, months	
Median	1.5
Range	0.2–104.6
Percentage of tumor involvement	
<25%	10 (19.2%)
≥25% and <50%	9 (17.3%)
≥50% and ≤75%	22 (42.3%)
>75%	11 (21.2%)
Number of lesion	
Solitary	19 (36.5%)
Uni-nodular (2–3)	33 (63.5%)
Multi-nodular (>3)	0 (0.0%)
Presence of extra-hepatic disease	
Yes	24 (46.2%)
No	28 (53.8%)
Portal vein and or IVC thrombosis	
Yes	23 (44.2%)
No	29 (55.8%)
BCLC stage	
C	51 (98.1%)
D	1 (1.9%)
Underlying liver disease	
Nil	13 (25.0%)
Hepatitis B	27 (51.9%)
Hepatitis C	1 (1.9%)
Multiple etiologies	11 (21.2%)
Prior treatment	
Nil	34 (65.4%)
Surgery	0 (0.0%)
Radiofrequency ablation	0 (0.0%)
TACE	14 (26.9%)
Sorafenib	0 (0.0%)
Others	1 (1.9%)
Multiple treatments	3 (5.8%)
Post-RT treatment	
Nil	26 (50.0%)
Surgery	0 (0.0%)
Radiofrequency ablation	0 (0.0%)
TACE	1 (1.9%)
Sorafenib	23 (44.3%)
Others	1 (1.9%)
Multiple treatments	1 (1.9%)
Child-Pugh score	
A5	20 (38.5%)
A6	12 (23.1%)
B7	12 (23.1%)
B8	6 (11.5%)
B9	2 (3.8%)
AFP level, nmol/L	
Mean	92979.6
Range	2.0–800000
Albumin, g/L	
Mean	33.4
Range	23–44
Bilirubin, umol/L	
Mean	23.00
Range	5–80
AST, U/L	
Mean	169.6
Range	0–689
ALT, U/L	
Mean	64.8
Range	10–228
ALP, U/L	
Mean	304.7
Range	79–1058
INR	
Mean	1.2
Range	1.0–1.5
Hemoglobin level	
Mean	11.6
Range	7.6–18.3
WBC, ×10^9^/L	
Mean	8.1
Range	3.5–20.1
Platelet, ×10^9^/L	
Mean	268.9
Range	60–895
Size of largest tumor (cm)	
Median, range	13.0 (3.2–24.0)
GTV volume (ml)	
Median, range	1692.8 (58.8–4696.7)
Normal liver volume (ml)	
Mean, range	2778.5 (822.5–5799.7)
Uninvolved liver volume (Normal liver – GTV) (ml)	
Median, range	1113.7 (0.00–1983.5)
Mean liver dose (Gy)	
Median, range	12.3 (6.7–17.2)

### Symptom response and duration

**Figure 1:**
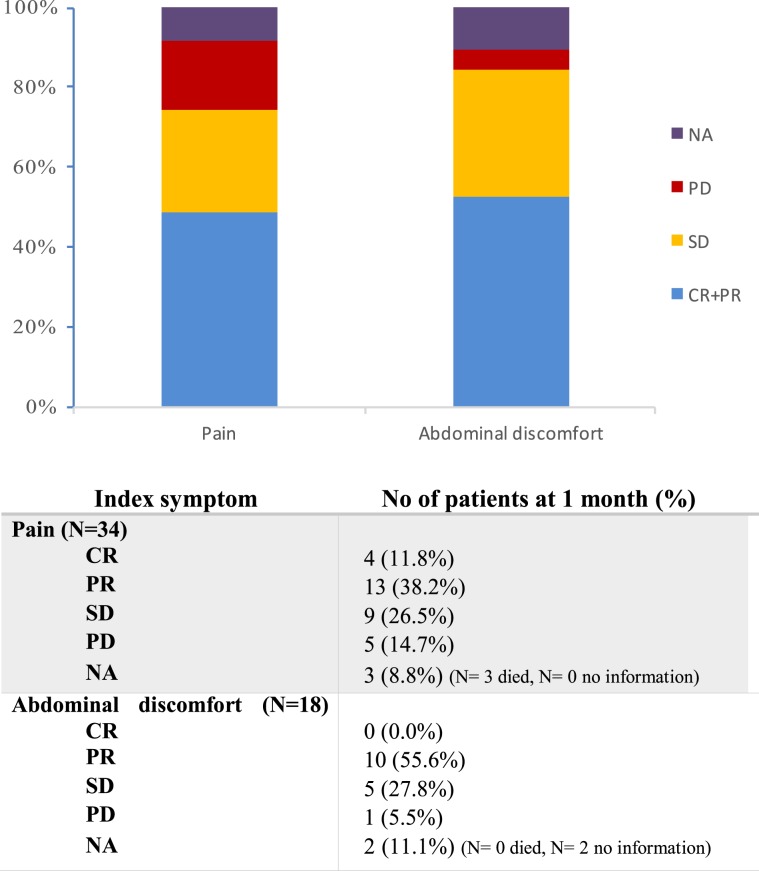
Bar Chart showing proportion of patients with clinical improvement of pain and abdominal discomfort at 1 month (CR + PR, SD, and PD).

The index symptom was pain in 34 patients (65.4%), and abdominal discomfort in 18 patients (34.6%). Figure 1 shows the percentage of patients who had improvement of index symptoms at 1 month. Overall, 51.9% (95% confidence interval Cl, 37.6–66.0%) of patients had clinical improvement of symptoms at 1 month; 50% (95% Cl, 30.8 – 78.5%) for patients with pain, and 55.6% (95% Cl, 32.4 – 67.6%) for those with abdominal discomfort. Five patients had missing information (n = 3, died before 1 month, n = 2, no information).

**Figure 2:**
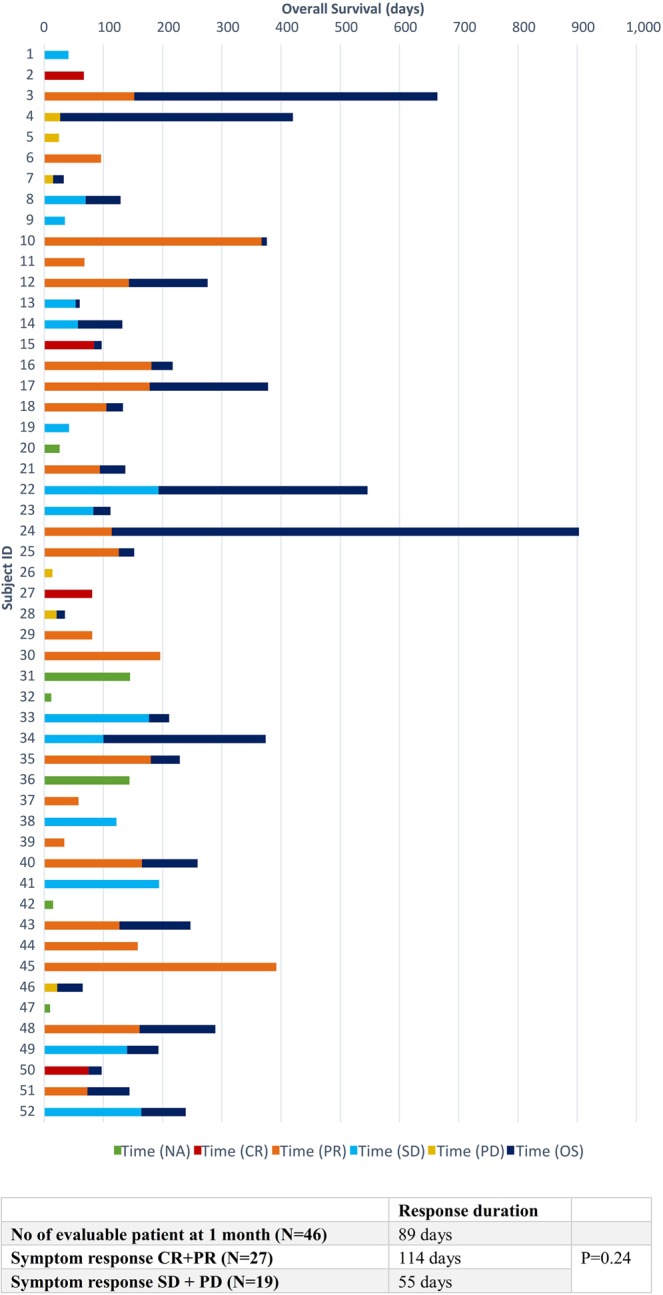
Symptom response, response duration, and overall survival of individual patient (N = 52).

Median time to symptom worsening was 89 days (range: 12–392 days). Patients with clinical improvement (CR + PR) at 1 month (median time 114 days, range: 34–392 days) had more durable control of symptoms than those without improvement (SD + PD) (median time 55 days, range: 14–194 days) (p = 0.24). Figure 2 shows the symptom response and response duration of individual patients. 21 patients (40%) did not experience worsening of symptoms before death. By means of the logistic regression model, no clinical or dosimetric factors could be identified in predicting symptom responders (Table 2).

**Table 2:** Uni-variate and multi-variate analysis in prediction of symptom response.

	Uni-variable		Multi-variable	
	Odds Ratio (95% CI)	P value	Odds Ratio (95% CI)	P value
Age	1.038 (0.984–1.095)	0.167		
Sex	0.986 (0.194–4.994)	0.986		
ECOG (0,1 vs 2)	2.000 (0.515–7.767)	0.317		
Hepatitis B	0.164 (0.082–1.528)	0.164		
Percentage of liver involvement	1.015 (0.992–1.038)	0.208		
Size of largest tumor	1.005 (0.878–1.150)	0.947		
Lesion number	0.462 (0.136–1.561)	0.214		
Presence of metastasis	1.373 (0.399–4.718)	0.615		
Portal vein thrombosis	0.563 (0.175–1.810)	0.335		
CP class	0.750 (0.226–2.491)	0.639		
Symptom: Pain vs. abdominal discomfort	0.729 (0.212–2.505)	0.615		

### Toxicity

Treatment is well tolerated with no grade 4 or 5 toxicities. The most commonly seen grade 3 toxicity was elevation of AST (n = 3, 5.8%). Grade 3 fatigue, nausea and vomiting was observed in 1 patient (1.9%). No patient developed classical RILD. 8.8% of patients had deterioration of CP score ≥ 2 at 3 months. Table 3 summarized the worst grade 3 or above toxicities.

**Table 3:** Worst Acute Toxicity CTCAE 4.0 ≥ grade 3.

	Grade 3	Grade 4–5
**Biochemical**		
Elevation of AST	3 (5.8%)	0 (0.0%)
Elevation of ALT	1 (1.9%)	0 (0.0%)
Bilirubin	1 (1.9%)	0 (0.0%)
Albumin	2 (3.8%)	0 (0.0%)
Platelet	1 (1.9%)	0 (0.0%)
WCC	0 (0.0%)	0 (0.0%)
Hemoglobin	0 (0.0%)	0 (0.0%)
Overall	7 (13.4%)	
**GI and others**		
Nausea and vomiting	1 (1.9%)	0 (0.0%)
Fatigue	1 (1.9%)	0 (0.0%)
Classical RILD	0 (0.0%)	0 (0.0%)
Cholangitis	0 (0.0%)	0 (0.0%)
Gastritis/ulcer	0 (0.0%)	0 (0.0%)
Hepatic failure	0 (0.0%)	0 (0.0%)
Overall	2 (3.8%)	
No of patients with deterioration of CP score ≥ 2 at 3 months without progression	3/34 (8.8%)	

### Survival, biochemical response, and radiological response

**Figure 3:**
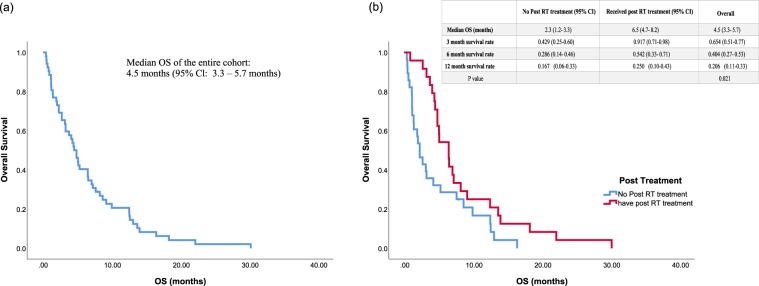
Kaplan Meier Curve (OS) of the (**a**) overall population from the time of radiotherapy; (**b**) Received subsequent therapy after RT vs. no further therapy.

With reference to Fig. 3, the median follow-up time was 4.7 months (range: 0.4 – 30.2 months); no patient was lost to follow-up. Overall, the median OS was 4.5 months (95% Cl: 3.3 – 5.7 months). Patients who received post-RT treatment had better OS (median: 6.5 months, 95% Cl, 4.7–8.2 months) than those receiving no further treatment (median: 2.3 months, 95% Cl, 1.2–3.3 months) (p = 0.021). Univariate and multivariate regression analysis in prediction of 90-day survival was shown in Table 4. Portal vein thrombosis (hazard ratio HR: 7.16, 95% Cl 1.41–36.32, p = 0.018) and baseline ALBI score (HR: 32.35, 95% Cl 4.23–36.32, p = 0.001) were identified as independent prognostic factors.

**Table 4:** Uni-variate and multi-variate analysis in prediction of 90-day survival.

	Uni-variable		Multi-variable	
	Odds Ratio (95% CI)	P value	Odds Ratio (95% CI)	P value
Age	0.958 (0.908–1.012)	0.125		
Sex	2.143 (0.467–9.839)	0.327		
ECOG (0,1 vs 2)	1.389 (0.401–4.806)	0.604		
Hepatitis B	4.364 (0.855–22.262)	0.076		
Percentage of liver involvement	1.004 (0.983–1.025)	0.714		
Lesion number	1.142 (0.605–2,156)	0.683		
Presence of metastasis	1.167 (0.358–3.797)	0.798		
**Portal vein thrombosis**	**4.182** (**1.241–14.096)**	**0.021**	**7.157** (**1.410–36.319**)	**0.018**
Baseline AFP level	1.390 (0.953–2.028)	0.087		
Baseline CP class	9.429 (0.966–92.0630	0.054		
**Baseline ALBI score**	**21.820** (**3.815–124.794)**	**0.001**	**32.353** (**4.231–36.319**)	**0.001**
**Post-RT Sorafenib**	**0.068** (**0.013–0.348)**	**0.001**	0.207 (0.029–1.490)	0.207

Among 35 patients had baseline elevation of AFP level, 17 patients (48.6%) had AFP response; notably, 7 of them did not receive further treatment after the RT. For 33 patients who had CT performed at 3 months, radiological response was observed in 5 patients (15.1%); 1 of them did not receive further treatment with CT images was presented in supplementary figure 1. Disease control was reported in 18 patients (54.5%).

## Discussion

This study supports the findings of previous phase II trial that palliative RT 8 Gy single fraction is an effective regime in relieving hepatic pain or abdominal discomfort in advanced HCC patients^15^. In the current study, substantial patients reported improvement of hepatic pain (50%) or abdominal discomfort (55.6%) at one month after RT. The median response duration was around 3 months.

Most advanced HCC patients experience symptoms of pain or abdominal discomfort at some point of their illnesses. Often, patients become refractory to available medical therapy for a period of time before they died and most of them suffered from severe symptom burden. Management of hepatic pain is challenging. Opioid is commonly prescribed in controlling cancer pain; however, in patients with liver function impairment, which is commonly seen in HCC patients, the parent drug and its metabolites may accumulate and precipitate encephalopathy and cause excessive sedation^17^. There is an unmet need for a better strategy to palliate pain and discomfort in the advanced HCC population.

There was a paucity of studies to evaluate palliative liver RT in the HCC population. Early studies of low-dose liver RT were mainly conducted in patients with liver metastases^7–14^. But it is known that patients with primary liver cancer have poorer prognoses, heavier symptom burden, worse systemic therapy response, but better tumor radio-sensitivity than those with metastatic liver diseases^18^. Also, most of these studies were conducted using obsolete RT techniques, long fractionation schedules, or with concurrent radio-sensitizers that are no longer used in routine clinical practice. The largest of these studies in liver metastases delivered 21 Gy in 7 fractions to the whole liver and randomized patients to misonidazole or placebo; there was an 80% response rate of abdominal pain, with a median duration of response of 13 weeks^13^. Another study observed a physician-reported symptom response of 54% at 2 weeks following 10 Gy in 2 fractions to the whole liver to palliate symptoms from liver metastases with two grade 3 toxicities^14^. In a recent prospective phase II trial by Soliman *et al*., patients with either primary or secondary liver cancers refractory to all therapies were treated with 8 Gy single fraction to whole liver^15^; there was clinically significant improvement at one month in 55% (95% Cl: 32–76%) of patients when assessing ‘average’ pain or discomfort, and in 59% (95% Cl: 32–76%) when evaluating ‘worst’ pain or discomfort in the past 24 hours. There was no difference in terms of symptom response between patients with HCC (n = 21) and liver metastases (n = 20). The benefits in terms of quality of life (QOL) improvement were comparatively modest. Overall, RT is well tolerated with only 7% of patients developed grade 3 toxicities.

The present study is unique in recruiting exclusively HCC patients among an endemic hepatitis B viral (HBV) infected population. HCC differ from other liver cancers with distinct disease course and treatment response; these patients are more often to have pre-existing chronic liver diseases and active viral infections, and are therefore more vulnerable to radiation-induced hepatic toxicities^5^. Of note, 73% patients in the present study are HBV carriers. 51.9% of patients had index symptom improvement at one month and a low rate of grade 3 toxicities, which concurs with previous studies. Among 34 patients alive at 3 months post-RT, only three (8.8%) patients developed progression of CP ≥ 2; it indicates that low dose radiation is generally safe even in a population of advanced disease and borderline hepatic function. We have commenced entecavir to all HBV carriers one week before RT and there were no viral re-activation observed.

Another strength of this study is that patients were prospectively followed regularly for their symptoms until their death. The median time to symptom worsening was around 3 months, which is similar to what was shown in earlier study with response duration of 13 weeks treated with more protracted fractionation. Also, similar response duration of 3 months was observed in patients with bone metastasis treated with RT of 8 Gy single fraction^19^. Using this shortest effective fraction is advantageous for advanced HCC patients with limited survival. The median OS of our cohort was only 4.5 months (95% Cl: 3.3–5.7 months); patients who had portal vein thrombosis (HR: 7.2, p = 0.01) or advanced ALBI grade (HR: 32.4, p = 0.001) had even poorer prognoses. Around 40% of the patients (n = 21) did not experience worsening of symptoms before death after the single fraction treatment. There was a trend suggesting that better symptom responders to RT also enjoy a better response duration (CR + PR 3.7 months vs. SD + PD 1.8 months, p = 0.24). Yet, we were unable to identify predictors of symptom responder.

Most patients (98%) in the present study had BCLC advanced disease in which Sorafenib is the current standard of care; however, Sorafenib had no benefit in prolonging patient’s time to symptomatic progression and there is limited data concerning its efficacy on symptom improvement^20,21^. Advanced HCC patients in our population often presented with pain or abdominal discomfort due to sizable tumors^22^. In our practice, low dose RT was used upfront for symptomatic patients to provide rapid palliation; Sorafenib was then started within 2 weeks after completion of RT. Among twenty-three patients treated with such approach, 52.6% had symptom improvement at 1 month and the median time of symptom worsening was 4 months, which compares favorably to the historical data of Sorafenib. There were no abnormal safety signals detected. Of note, the symptom response and toxicity measurement of RT in this group of patients might have been confounded by the effect of Sorafenib. Whether combining low-dose RT to Sorafenib in advanced symptomatic HCC patients would confer quality of life (QoL) benefit warrants further exploration.

Few patients had marker or radiological response after RT alone, and the responses were usually short-lived. Indeed, the radiation dose (EQD_2_ 2 Gy equivalent dose = 12 Gy) is too low to expect tumor response. Thus, the palliative effect is unlikely due to tumor shrinkage, but its exact mechanism remains unclear. Previous studies in bone metastases postulated that radiation might reduce the tumor-secreting cytokines that sensitize patients to pain signals^23,24^. Further translational research to correlate the change of cytokines levels to pain response after low-dose liver RT would be of interest.

Nonetheless, our study has several limitations. This is a single institution retrospective study. We have included consecutive patients treated with low-dose liver RT in our department during the study period into analysis to minimize any selection bias; the symptom response information was prospectively collected resulting in a low rate of missing information. However, there was no comparative group treated with medical palliative therapy alone to determine whether RT had any added benefit over medical symptoms management. Also, as the primary endpoint of symptom improvement was measured by physicians’ reported outcomes, potential bias might have been introduced. Furthermore, sequential evaluation on the time and pattern of progression was not otherwise feasible as patients’ limited survival has precluded serial imaging. On the other hand, our study offers the advantage of consistent treatment facilities and personnel with multidisciplinary discussions and team-based, protocol-oriented treatment decisions within a single institution. In addition, treatment response was documented prospectively adhering to unified criteria as defined in our institutional protocol. Although we could not completely eliminate physician bias or individual variability, our results are hypothesis generating and have stemmed from preliminary results suggesting promising RT effects among the advanced HCC population with unmet clinical needs, in which we hope to shed light on future clinical research. More robust tools such as the brief pain inventory (BPI), Edmonton Symptom Assessment Scale (ESAS), or MD Anderson Symptom Inventory could be utilized in our future prospective study^25–27^. Collection of patient reported outcomes (PRO) and quality of life (QOL) data would also be employed for a more comprehensive symptom assessment^28^.

To date, there is very limited data to recommend any effective palliative treatment among the terminal HCC population. Our preliminary results suggest that palliative low-dose liver RT may potentially be a safe, effective, and convenient treatment in advanced HCC patients with pain or abdominal discomfort. Our findings within the Asian population are consistent with that among the Western population. Taken together, this provides solid ground for potential global prospective randomized phase III trials.

## Supplementary information

Supplementary materials.
