# Supplementary material for: Palliative Liver Radiotherapy (RT) for Symptomatic Hepatocellular Carcinoma (HCC)
Source: Sci Rep. 2020 Jan 27;10:1254. doi: 10.1038/s41598-020-58108-1 (PMC6985173; doi:10.1038/s41598-020-58108-1)

Palliative Liver Radiotherapy (RT) for Symptomatic Hepatocellular Carcinoma (HCC)

Running title: Palliative liver radiotherapy for hepatocellular carcinoma

Cynthia SY Yeung*[1], CL Chiang*[1,2,3], Natalie SM Wong[1], SK Ha[2], KS Tsang[2], Connie HM Ho[1], B Wang[3], Venus WY Lee[1], Mark KH Chan[4], Francis AS Lee[1]

1. Department of Clinical Oncology, Tuen Mun Hospital, Hong Kong (SAR)
2. Department of Clinical Oncology, University of Hong Kong, Hong Kong (SAR)
3. Department of Clinical Oncology, HKU-Shenzhen Hospital, China
4. Department of Radiation Physics, Imperial College London NHS Healthcare Trust, Charing Cross Hospital, U.K.

*** Co-first author**

Supplementary Figure 1: CT images showing good radiological response after receiving palliative liver RT 8Gy single fraction

Baseline

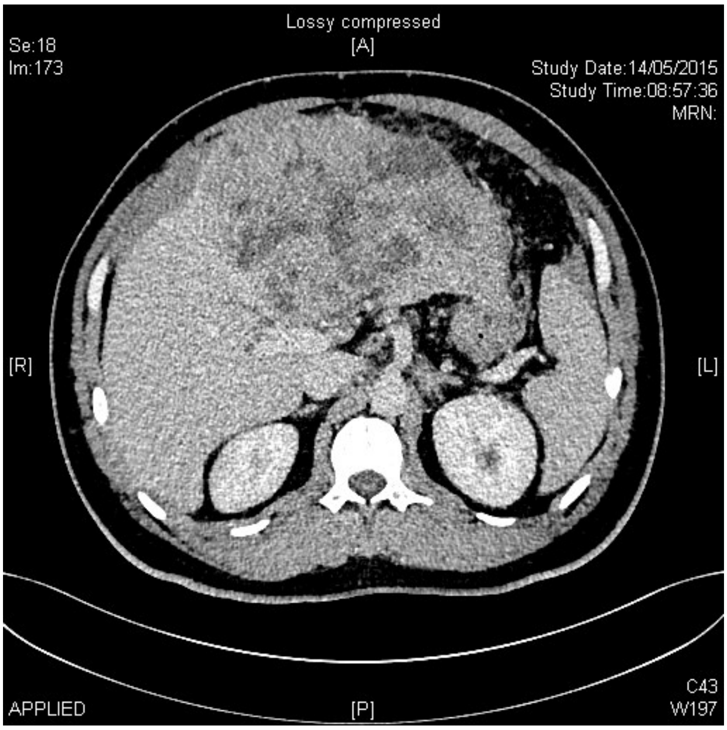

3 months after radiotherapy

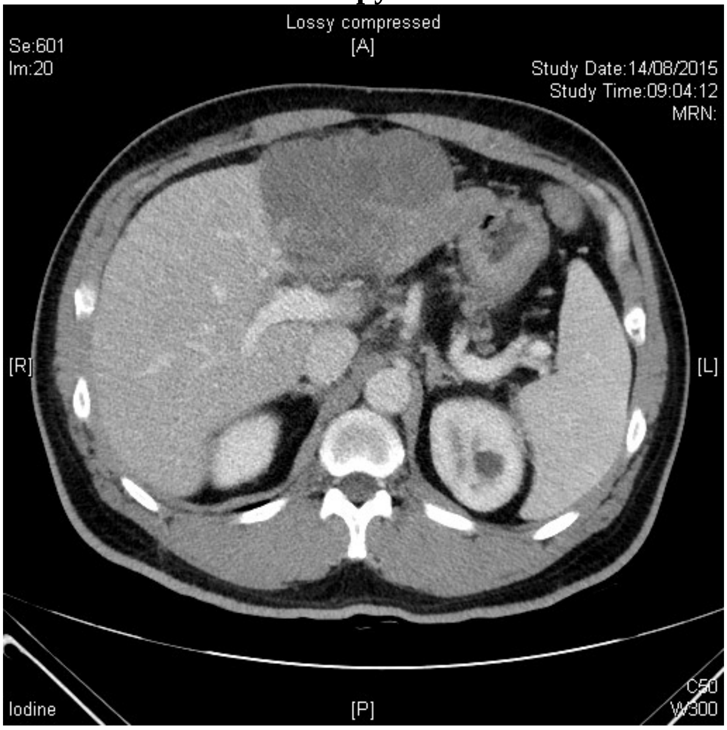

Supplement: Supplementary file 1 — Supplementary materials. [file 41598_2020_58108_MOESM1_ESM.pdf]
